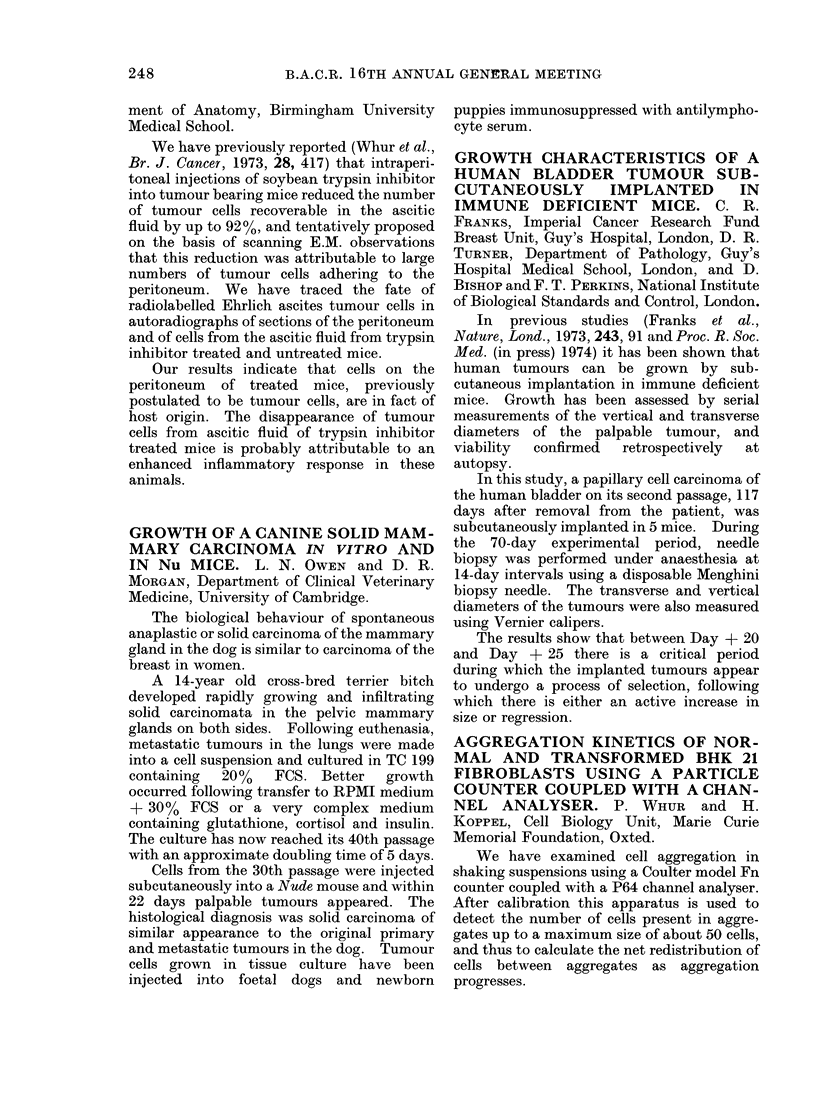# Proceedings: Growth of a canine solid mammary carcinoma in vitro and in Nu mice.

**DOI:** 10.1038/bjc.1975.181

**Published:** 1975-08

**Authors:** L. N. Owen, D. R. Morgan


**GROWTH OF A CANINE SOLID MAMMARY CARCINOMA *IN VITRO* AND IN Nu MICE.** L. N. OWEN and D. R. MORGAN, Department of Clinical Veterinary Medicine, University of Cambridge.

The biological behaviour of spontaneous anaplastic or solid carcinoma of the mammary gland in the dog is similar to carcinoma of the breast in women.

A 14-year old cross-bred terrier bitch developed rapidly growing and infiltrating solid carcinomata in the pelvic mammary glands on both sides. Following euthenasia, metastatic tumours in the lungs were made into a cell suspension and cultured in TC 199 containing 20% FCS. Better growth occurred following transfer to RPMI medium + 30% FCS or a very complex medium containing glutathione, cortisol and insulin. The culture has now reached its 40th passage with an approximate doubling time of 5 days.

Cells from the 30th passage were injected subcutaneously into a *Nude* mouse and within 22 days palpable tumours appeared. The histological diagnosis was solid carcinoma of similar appearance to the original primary and metastatic tumours in the dog. Tumour cells grown in tissue culture have been injected into foetal dogs and newborn puppies immunosuppressed with antilymphocyte serum.